# Antioxidant, Heavy Metals and Elemental Analysis of *Holoptelea integrifolia* Planch

**DOI:** 10.4103/0250-474X.45419

**Published:** 2008

**Authors:** A. Saraswathy, S. Nandini Devi, D. Ramasamy

**Affiliations:** Captain Srinivasa Murti Drug Research Institute for Ayurveda and Siddha (CCRAS), Anna Hospital Campus, Arumbakkam, Chennai-600 106, India

**Keywords:** Stem bark, *Holoptelea integrifolia*, antioxidant activity, α-tocopherol, ethanol extract

## Abstract

The ethanol crude extract of stem bark of *Holoptelea integrifolia* Planch. traditionally used in Indian system of medicine was screened for its antioxidant activity using α-tocopherol as standard antioxidant. The free radical scavenging potential of the extract was evaluated by two different antioxidant methods; ferric thiocyanate and thiobarbituric acid method. The ethanol extract was found to exhibit good antioxidant property. Further physicochemical constants, elemental and heavy metal analysis of stem bark have been described.

Reactive oxygen species (ROS) are a class of highly reactive molecule derived from the metabolism of oxygen. ROS, including superoxide radicals, hydroxyl radical and hydrogen peroxide molecule, are often generated as by product of biological reactions or from exogenous factors. There is extensive evidence to implicate ROS in the development of degenerative diseases^1^.

Free radicals have been implicated in causation of ailments such as diabetes, liver cirrhosis, and hepatotoxicity^2^. These by products inactivate enzymes and damage important cellular components causing tissue injury through covalent binding^3^, thus have been shown to augment collagen synthesis and fibrosis. The increased production of toxic oxygen derivatives is considered to be a universal feature of stress conditions.

The body produces several antioxidant enzymes, including superoxide dismutase (SOD), catalase and glutathione peroxidase that neutralize many types of free radicals. In addition to enzymes, many vitamins and minerals such as vitamin C, vitamin E, beta-carotene, lutein, lycopene, vitamin B2, and cysteine acts as antioxidant. Many herbs such as bilberry, turmeric, grape, orange, tea and pine bark also provide antioxidant property.

Several antioxidant based formulations have been developed for the treatment of diseases like atherosclerosis, stroke, diabetes, Alzheimer's disease and cancer during the last 3 decades^4^. This has attracted a great deal of research interest in natural antioxidants. It is necessary to study the botanicals to screen out for their antioxidant potential. Hence in this report we investigated the ethanolic extract of the bark of *Holoptelea integrifolia* Planch for antioxidant activity, as review of literature afforded no information on the antioxidant potential of the plant. *Holoptelea integrifolia* Planch. (Syn. *Ulmus integrifolia* Linn., *Chirabilva/Poothigam*) belonging to the family Ulmaceae^5^ is a medium sized to large deciduous tree, whitish or yellowish grey bark in irregular flakes when freshly cut; leaves simple, alternate, elliptic, entire, glabrous with rounded (or) cordate; flowers greenish yellow racemes or fascicles; fruits sub orbicular samara. The tree is distributed throughout India in decidous forests^6^. The chemical constituents of stem bark are friedelin, friedelin-3-β-ol, β-sitosterol, hederagenin (heart wood), hexacosanol, fatty acid esters, holoptelin A and B and β-amyrin (leaves). Bark has many medicinal properties and used in tuberculosis, piles, fistula, abdominal diseases, leprosy, polyuria, vomiting and rheumatism swelling^7^,^8^. Pre-clinical studies in rats shows that the drug significantly reduces the body weight thereby proving its antiobesity properties^9^. It is also used in Ayurvedic formulation like *Piyusavalli Rasa, Gandharva hastadi kwata curna*^10^.

The chemicals used were of analytical grade. Linoleic acid, phosphate buffer, ammonium thiocyanate were purchased from SRL Chemicals, Chennai, Ferrous chloride was procured from Central Drug House (P) Ltd, New Delhi, Hydrogen peroxide was procured from Chemspure, Chennai and Trichloroacetic acid, Thiobarbituric acid, Vitamin E were purchased from S. D. Fine Chemicals, Mumbai.

Stem bark of *Holoptelia integrifolia* was collected from Chennai, and identified with the help of Flora of the Presidency of Madras^11^, by the botanist at CSMDRIA (CCRAS), Arumbakkam, Chennai (voucher specimen no. L/245).

The stem bark was shade dried, powdered and stored in an air tight container at 27°. Ten grams of powder was accurately weighed and exhaustively extracted by absolute alcohol using Soxhlet apparatus. The extract was filtered, evaporated to dryness under vacuum (205 mg) and used for the antioxidant activity studies.

Ferric thiocyanate method is based on the determination of peroxide (lipid) at the primary stage of linoleic acid peroxidation. The peroxide reacts with ferrous chloride to form a reddish ferric chloride pigment which is measured at 500 nm. The standard method as described by Kikuzaki and Nakatani^12^ was used. A mixture of 4 mg of sample in 4 ml of 99.9% ethanol (200 μg/ml), 4.1 ml of 2.52% linoleic acid in 99.9% ethanol, 8 ml of 0.05 M phosphate buffer (pH 7.0) and 3.9 ml of water were taken in a screw capped vial and was incubated at 40° in the dark. To 0.1 ml of this solution, 9.7 ml of 75% ethanol and 0.1 ml of 30% ammonium thiocyanate were added. Precisely 3 min after the addition of 0.1 ml of 0.02 M ferrous chloride in 3.5% hydrochloric acid to the reaction mixture, the absorbance was measured at 500 nm by UV spectrophotometer (Perkin Elmer, Lambda EZ201) for every 24 h until the absorbance of the control reached maximum. The control and the standard were subjected to the same procedure as the sample except that for the control, only the solvent was used, and for the standard, 4 mg of the sample was replaced by 4 mg of vitamin E (200 μg/ml).

The formation of malonaldehyde is the basis for the TBA method as reported by Ottolenghi^13^ used for evaluating the extent of lipid peroxidation. At low pH and high temperature (100°), malonaldehyde binds TBA to form a red complex and its absorbance is measured at 532 nm. Two millilitres of 20% trichloro acetic acid and 2 ml of 0.67% TBA solution were added to 1 ml of the mixture containing the sample prepared in the FTC method and incubated similarly. This mixture was kept in a water bath (100°) for 10 min. After cooling to room temperature, it was centrifuged at 3000 rpm for 20 min. Absorbance of the supernatant was measured at 532 nm. Antioxidant activity was based on the absorbance on the final day of the assay. The control and standard vitamin E (4 mg) were also subjected to similar procedure.

Analytical data like total ash, acid insoluble ash, water soluble ash, n-hexane, water and alcohol soluble extractives, loss on drying at 105° were carried out as per WHO guidelines^14^. Minerals and heavy metal analysis were carried out by atomic absorption spectroscopy^15^ (Perkin Elmer-400, carrier gas was argon and the flow rate was 2 ml/3 min).

Accurately weighed 500 mg of stem bark powder was taken in round bottom flask. To this 5 ml of conc. nitric acid was added and refluxed for half an hour in a hot plate at 80-100°. It was then cooled. Five millilitres of concentrated nitric acid was added and warmed on water bath. Two millilitres of 30% hydrogen peroxide solution was added to the above mixture and warmed for 10 min till clear solution was obtained. It was then cooled, filtered through Whatman filter paper No. 42 and made up to 100 ml using deionised water.

The antioxidant activity of ethanol extract of the stem bark of *Holoptelia integrifolia* was assessed by both FTC and TBA methods at a concentration of 0.02% and compared with vitamin E. In FTC method, the amount of peroxide at the initial stage of lipid peroxidation was determined. During the linoleic acid oxidation, peroxides are formed, which oxidize Fe^+2^ to Fe^+3^. The formed Fe^+3^ ions complexes with thiocynate ions (SCN^−^), which has a maximum absorbance at 500 nm. The concentration of peroxide decreases as the antioxidant activity increases. Lower the absorbance value exhibited higher the antioxidant activity. Vitamin E showed an initial absorbance of 0.07 and a maximum absorbance of 0.28 on day 5. Extract had absorbance value of 0.083 initially and gradually raised to 0.29 on day 5. The control had the highest absorbance value 0.83, followed by extract (0.29), standard vitamin E (0.28) on day 5. Based on the results obtained, the extract was found to possess antioxidant activity, which is comparable to standard vitamin E, at a concentration of 4 mg.

The absorbance values from TBA method indicated the total peroxide values produced by the oxidation of linoleic acid. The librated malonaldehyde formed a red complex with TBA. The increase of the amount of red pigment formed correlates with oxidative rancidity of the lipid. Higher absorbance values indicate lower level of antioxidant activity. The control had the highest absorbance value (0.35), followed by *Holoptelea integrifolia* (0.08) and Vitamin E (0.075). The present study reveals that *Holoptelia integrifolia* showed antioxidant property which is comparable to the standard vitamin E. Thus ethanol extract exhibited significant *In vitro* antioxidant activity by inhibiting the oxidation of linoleic acid in both FTC and TBA methods. The activity was comparable with standard vitamin E.

Physicochemical data of the stem bark of *Holoptelea integrifolia* is tabulated (Table 1). This analysis showed ash content of 11.75% and water soluble ash of 10.5% indicating the presence of inorganic matter. Acid insoluble ash 0.36% shows the presence of silicates in the bark. Water soluble extractive value (10.52%) is due to the presence of sugars, acids, polar constituents, glycosides of steroid, alkaloids and coumarines. n-Hexane soluble extractive value (0.62%) reveals the presence of less polar straight chain compounds and waxy materials. Alcohol soluble extractive value (2.18%) shows the presence of fewer amounts of polar substances like phenols, tannins, glycosides and flavonoids in the stem bark. These physicochemical data would help for the identification of the drug from its substitutes/adulterants.

**TABLE 1:** PHYSICOCHEMICAL DATA OF *HOLOPTELEA INTEGRIFOLIA*

Parameters	values±SD
n-Hexane soluble extractive (% w/w)	0.62±0.04
Alcohol soluble extractive (% w/w)	2.18±0.56
Water soluble extractive (% w/w)	10.52±0.35
Total ash (% w/w)	11.75±0.08
Water soluble ash (% w/w)	10.52±0.35
Acid insoluble ash (% w/w)	0.36±0.06
Alkalinity of water soluble ash (ml of 0.1N HCl)	0.29±0.05
Loss on drying at 105°(% w/w)	10.06±0.07

The data is presented as mean±SD, n=3

Heavy metals viz. lead (0.11), cadmium (0.03) and mercury (0.001) of the stem bark was found to be within the permissible limits as per WHO guidelines (Table 2). The stem bark was free from arsenic, thereby proving the safety of its utilization in Ayurveda and Siddha systems. Mineral elements such as iron (2.17), copper (0.05), manganese (0.08), zinc (0.93), nickel (0.02), cobalt (0.11), chromium (0.13) are found in considerable amount which may be directly or indirectly helpful in the management of many diseases. Thus the results of the present study support the view that some traditionally used Indian medicinal plants particularly the stem bark of *Holoptelea integrifolia* are promising source of potential antioxidants.

**TABLE 2:** HEAVY METAL AND MINERAL CONTENT ANALYSIS OF *HOLOPTELEA INTEGRIFOLIA*

Heavy Metals and Mineral contents	Value (ppm)
Cadmium	0.03
Lead	0.11
Mercury	0.001
Arsenic	0.00
Iron	2.17
Copper	0.05
Manganese	0.08
Zinc	0.93
Nickel	0.02
Cobalt	0.11
Chromium	0.13

Average of 3 determinations
